# An Apple-watch all day keeps your life astray—or does it?

**DOI:** 10.1177/14034948251370466

**Published:** 2025-09-04

**Authors:** Eivind Meland

**Affiliations:** Department of Global Public Health and Primary Care, Bergen, Norway

**Keywords:** Self-tracking technology, self-preoccupation, motivation, mindfulness

## Abstract

**Aims:**

This paper discusses whether self-monitoring technology for continuous self-evaluation may harm us as individuals and communities. The aspiration of obtaining absolute knowledge is spoken of in *Genesis*. The story of the fall is a basic and universal human myth that warns against the aspiration to acquire absolute knowledge. Is self-evaluation a recipe for being alienated from ourselves and from others or may it serve as motivation for needed change?

**Conclusions:**

**Changes in western culture within and outside healthcare indicate that self-evaluation can be balanced and complemented by new common ground in medical ethics and psychotherapy. Promoting medical functionalism, as suggested by the United Nations World Social Report, is a step in this direction. Practices allowing open presence and mindfulness and reframing of the health concept can also pave the way for stronger solidity and robustness in individual people combined with communal engagement and responsibility.**

## Advances in self-tracking technology and mentality

By 2016, more than 160,000 medical and health-related technologies were available on the market, allowing people to monitor their behavior and physical conditions themselves. Wearable devices such as the Apple Watch and online programs can interpret biological, physical and mental changes for self-reflection and sharing with others. Social media platforms also allow groups of people to compare their results and experiences. This technology has influenced how people think about themselves—a trend that some medical sociologists call ‘the quantified self’ [1].

Strong supporters of these technologies even encouraged a change in medical ethics, where ‘P4 medicine’ or ‘personalized medicine’ would become more common in medical practice. This model includes four ideas: personalized, predictive, preventive and participatory [2]. It was expected that, within 10 years, this broader perspective—linking communities and biology—would help us understand disease better and allow blood to act as a window into a person’s health.

## Does it work and what about side-effects?

The effects of these technologies have been studied, with some positive results in randomized controlled trials (RCTs) and in meta analyses [3–5], especially when other clinical support was also provided [6,7]. However, other studies showed little or no benefit [8]. For example, some new glucose-monitoring tools helped control blood sugar but also increased the risk of side effects [5]. Weight control may improve, but it could also slightly raise scores linked to eating disorders [9].

Many studies did not properly monitor negative effects, especially long-term psychological problems. Still, some studies did report positive mental health results [3,10]. The benefits may last for only a short time and tend to fade over time [11]. These tools may work best for people who are well educated and have good health knowledge and motivation [12], although some studies show that people with less digital access can also benefit [13]. Long-term observation studies have found that worrying about body size can have unexpected effects—sometimes making weight problems worse and lowering people’s self-esteem and subjective health [14].

A recent, careful meta-analysis says we still do not know enough about:

1. how users think and feel,
2. the possible negative sides of self-tracking,
3. the effects on society and
4. how it changes the healthcare system and its workers [12].

These concerns match Deborah Lupton’s warning that self-tracking could be forced on people, putting private data in the hands of insurance companies and other powerful groups [1]. Still, research shows we may be seeing medical improvements, with short-term benefits—although not without risks.

## The illusion of certain knowledge

It is hard, or even impossible, for modern medical research to fully understand how technology changes how people see themselves and what they value as a society. We rarely question that we are part of a long history of ideas, where we have made important decisions about how we understand disease, life and what it means to be human. In ancient and medieval times, medicine balanced trust (faith) with experience-based treatment [15]. Faith and trust were part of healing. The word ‘patient’ itself comes from the idea of ‘trusting patience.’ Over time, modern medicine moved away from this and started focusing only on objective, scientific views of illness. Disease came to be seen as something we could completely understand. This belief, some say, repeats the mistake made by the first humans in the Garden of Eden—thinking we could know everything.

At the same time, as science progressed, our idea of nature and creation as something sacred was replaced with the view of nature as a resource to be used. Faith in being part of nature faded [16]. Faith was removed from man’s experience of being embedded in nature and creation. Philosopher Charles Taylor (1931–2024) described this as a change from a ‘porous self’ (open to the world) to an ‘encapsulated self’ (closed and individual). Trust in nature and relationships was replaced by focus on the self [17].

We are now seeing signs of crisis in medicine: lack of staff and resources, growing expectations from healthy people and more young people getting questionable medical labels. These problems may come from a narrow view of health and illness. People are not just biological objects—they seek meaning and understanding. When medicine treats them like objects, they often push back. Mistakes in how we think about knowledge faced with individual people can lead to bigger problems in society as a whole [18].

## Critical voices

Some researchers believe that after World War II (WWII), capitalism changed its focus, from controlling other countries to creating markets based on people’s physical and mental health. They say this shift was part of how capitalism kept going, through constant change and innovation [19]. In the latter paper, the authors quote Vannevar Bush, who led the United States Office of Scientific Research and Development during WWII. He said that new ‘frontiers of the mind’ could bring progress and a better life if explored with the same boldness used in war. According to the authors, basic science was seen as a way to fight disease, protect national security and grow the economy—shaping health not around patients’ real experiences [20], but around medical expansion. Ivan Illich warned that this way of thinking suggested that perfect health could only be reached by eliminating all disease [21]. Karlsen and Strand argue that this is just the beginning. There is no clear set of ‘ethical issues’ or even a ‘slippery slope’ that can be avoided, only good intentions. Therefore, we are seeing a constant push to turn new problems into market opportunities [19].

Other critics have pointed out that high-tech personalized medicine (such as P4-medicine) is different from person-centered medicine, which focuses more on people’s lives and stories [22]. Like Illich, they worry that turning social or personal problems into medical ones can distort our understanding, reducing complex issues to biology alone.

## Alternative ways of seeing and acting

Some of these criticisms may seem alarmist, but they can help us reflect. Life will always include outside pressures. What matters is how we respond—by developing inner motivation through meeting basic human needs like relations, skills and autonomy [23]. There are signs of change in both society and healthcare. These suggest we can find a better balance—between diagnosing disease and supporting people’s ability to live well [24], and between the limits of technology and our natural ability to heal. The United Nations has encouraged a shift in health priorities: from fighting disease to improving how people function in daily life [24]. Danish philosopher and theologian Knud E. Løgstrup (1905–1981) promoted being fully present in the moment. He believed that logical thinking alone is not enough for healing—we also need trust and appreciation for life, which anyone can access, regardless of belief [25].

But Løgstrup also criticized religious groups for focusing too much on salvation and the afterlife. This, he argued, weakened the importance of compassion and caring for others here and now. Like Charles Taylor [17], he encouraged cooperation between religious and non-religious people to limit over-medicalization and promote both inner strength and community responsibility.

This way of thinking is now seen in Western practices influenced by Eastern ideas, such as *mindfulness* and *open presence*. These focus on being aware of your breathing and staying present. In this context, spirituality means being here and now—aware and attentive.

### Mindfulness and communal responsibility

Mindfulness became important for health professionals after a 1999 article in *JAMA* [26]. Later studies from Norway, the United States and Spain showed that it can improve the mental health of healthcare workers and the quality of their interactions with patients [27–30]. A meta-analysis found that the effects of mindfulness are not always clear, partly because the term means different things to different people [31]. Ronald M. Epstein (b. 1956)—a key figure in this field—recently defined mindfulness as ‘a practice of being present. In medicine, it means paying calm and non-judging attention to yourself and others, with the goal of acting clearly, wisely, and with compassion.’ He stressed the importance of presence, curiosity and a beginner’s mindset [32].

Although debated, mindfulness seems possible to use at a community level—for example, in schools in low-income areas in the United States, where it has helped reduce problems [33]. Still, mindfulness is sometimes criticized. The Western version often turns a deep religious practice into a product for personal wellbeing [34]. Instead, it should be a starting point for social responsibility and communal commitment.

Science writer Tor Nørretranders (b. 1955) discusses how mindfulness can support better social interaction and help renew society and democracy [35]. He distinguishes between empathy and compassion. Empathy can wear us out and make us focus on ourselves. Compassion, on the other hand, can be trained—allowing us to help others kindly, without burning out or passing stress on. Mindfulness, then, is not about personal happiness alone. It is a way to grow inner strength while fulfilling our social responsibilities.

## Concluding remarks

Constant self-monitoring, comparison with others and trying to meet outside standards can make us feel like we are never enough. This can lead us to believe that happiness comes from consuming healthcare or products. But Løgstrup reminds us to start with *gratitude*—not for success, but as a basic attitude toward life and others. Mindfulness, practiced with kindness, can help build this attitude. Nørretranders believes that kindness and compassion are essential—not just for personal healing, but also to restore unity, dignity and trust in society. Could this be a new direction for medicine and public health, and the wider society as well?
